# Development and Psychometric Validation of the Human Papillomavirus Prevention Health Literacy Scale for College Students in Taiwan

**DOI:** 10.1097/jnr.0000000000000767

**Published:** 2026-08-19

**Authors:** Ching-Yi LAI, Shu-Chen YANG, Chieh-Yu LIU, Hung-Ru LIN, Yuan-Mei LIAO, Tzu-Ying LEE

**Affiliations:** 1 Department of Nursing, Central Taiwan University of Science and Technology, Taichung, Taiwan; 2 Institute of Community Health Care, College of Nursing, National Yang Ming Chiao Tung University, Taipei, Taiwan; 3 School of Nursing, National Taipei University of Nursing and Health Sciences, Taipei, Taiwan; 4 Institute of Clinical Nursing, College of Nursing, National Yang Ming Chiao Tung University, Taipei, Taiwan

**Keywords:** human papillomavirus, HPV, college students, health literacy, scale

## Abstract

**Background:**

Human papillomavirus (HPV) is a common sexually transmitted virus associated with several types of cancer. Because college students are a high-risk group, understanding their health literacy with regard to HPV prevention is critical. However, no suitable measurement tools are currently available for this purpose.

**Purpose:**

In this study, an HPV Prevention Health Literacy Scale (HPV-HLS) was developed, and its reliability and validity were assessed.

**Methods:**

A cross-sectional design was used, and the developed scale was validated on a sample of 372 college students from two universities in northern Taiwan and one university in central Taiwan. The initial scale comprised 49 items, developed from qualitative interviews and a literature review. After content validity assessment and item analysis, the scale was refined to 37 items. Exploratory factor analysis (EFA) was conducted with 150 participants, followed by confirmatory factor analysis (CFA) with 222 participants. Concurrent validity and internal consistency reliability were assessed. Data were collected from November 2021 to December 2022.

**Results:**

The results of the EFA revealed a two-factor model accounted for 60.30% of the total variation, and those of the CFA demonstrated favorable goodness-of-fit indices (GFI = .81, CFI = .92, TLI = .91, RMSEA = .07, and SRMR = .02). Moreover, the average variance extracted (AVE) for the two factors were .56 and .64, respectively, with corresponding composite reliability (CR) values of .96 and .93, indicating excellent convergent validity. Notably, the AVE surpassed the squared product-moment correlation of the corresponding factors, affirming good discriminant validity. The final iteration of the HPV-HLS comprised 21 items with two subscales: comprehension and evaluation of general information and seeking information from health care professionals. Concurrent validity was found between the proposed scale and the Mandarin Multidimensional Health Literacy Questionnaire (*r* = .68, *p* < .001). The HPV-HLS demonstrated good internal consistency (Cronbach’s α = .959).

**Conclusions/Implications for Future Practice:**

After rigorous psychometric testing, the newly developed HPV-HLS demonstrated excellent reliability and validity, establishing it as an objective tool for evaluating HPV prevention health literacy among college students. This HPV-HLS may be used by health care providers and educators to assess health literacy and evaluate the effectiveness of clinical interventions to prevent HPV infection.

## Introduction

Human papillomavirus (HPV) infection is the most prevalent sexually transmitted disease in the world (Centers for Disease Control and Prevention [CDC], 2024a). With over 200 types (Ahmed et al., 2023), low-risk HPVs can cause warts, while high-risk HPVs are linked to oropharyngeal, head and neck, cervical, vaginal, vulvar, penile, and anal cancers (CDC, 2024a). At present, an estimated 831,204 new cancer cases and 422,935 cancer deaths worldwide are attributable to HPV infection, making HPV infection prevention a crucial public health priority (J. Zhang et al., 2025).

HPV infection primarily spreads through sexual activity (Ahmed et al., 2023). Many sexually active individuals are susceptible to infection, and those who are infected often remain unaware, as most infections exhibit no visible signs or symptoms (CDC, 2024b). Risk of HPV infection escalates significantly with the number of sexual partners an individual has over their lifetime (Lee et al., 2024), with those having one sexual partner facing a 58.4% risk and those with 3–6 partners facing a 90.9% risk of HPV infection (Forman et al., 2012). As individuals aged 15–24 years have the highest incidence of HPV infection (Chesson et al., 2021), college students (typically 18–24 years old) are in this high-risk category. The independent and liberated lifestyles of college students often lead to their engaging in active sexual relationships, which can involve risky behaviors such as having multiple sexual partners and not taking protective measures against sexually transmitted infections (Yin et al., 2021), significantly increasing their vulnerability to HPV infection.

The risk of HPV infection may be mitigated through primary prevention strategies. These include practicing safe sexual behaviors such as abstaining from having multiple simultaneous sexual partners and correctly using condoms throughout sexual intercourse (CDC, 2024b). In addition, sexually active women should undergo regular HPV-related screenings such as pap smear tests and HPV DNA tests (World Health Organization, 2021). Both men and women can receive the HPV vaccine (CDC, 2024a). Despite the availability of these options, research findings indicate college students often lack sufficient knowledge about sexually transmitted diseases. Furthermore, students are often unable to assess the accuracy of disease-related and prevention-related information they receive (Lederer & Sheena, 2021). Thus, without access to appropriate and accurate sexual health resources, they may struggle to maintain healthy sexual relationships, which can negatively impact their health and increase their risk of HPV infection (Lechner et al., 2013).

The ability of college students to adopt effective preventive measures against HPV infection is closely linked to their health literacy (Johnson et al., 2022). The Office of Disease Prevention and Health Promotion (2020), in Healthy People 2030, defines health literacy as “the degree to which individuals can find, understand, and use information and services to inform health-related decisions and actions for themselves and others.” Nutbeam (2000) proposed three levels of health literacy: functional, critical, and interactive. Functional health literacy underscores the importance of having the literacy skills necessary to comprehend basic health information (Nutbeam, 2000). Critical health literacy describes the capacity to analyze health information, which is essential to assessing credibility independently and making informed health decisions (Ogbadu-Oladapo et al., 2024). Interactive health literacy emphasizes the communication and social skills critical to productive interactions with health care professionals and others, including family and friends (Nutbeam, 2000). Over the past decade, most published studies have focused on health literacy in the context of chronic disease management (Jang et al., 2024). Although the role of health literacy in infectious disease prevention, particularly human immunodeficiency virus (HIV) and COVID-19 prevention (Forsythe, 2020; Istiko et al., 2024), has been growing, its application in HPV prevention remains limited. Unlike COVID-19 and HIV, HPV infections are often asymptomatic and predominantly affect younger populations. Thus, the progression and public perception of HPV and HIV are markedly different. HIV typically advances to symptomatic stages, prompting medical intervention, whereas HPV infections frequently remain undetected for years, potentially progressing to cervical or other cancers without noticeable symptoms (CDC, 2024b). Moreover, HPV prevention relies heavily on vaccination and routine screenings (e.g., Pap smears) to detect precancerous changes (CDC, 2024a). In contrast, HIV prevention focuses primarily on safe sexual practices and pre-exposure prophylaxis (Istiko et al., 2024). These distinct features highlight the need for a targeted assessment tool to evaluate HPV prevention health literacy.

Even though several common health literacy measurement and assessment tools exist, including the Rapid Estimate of Adult Literacy in Medicine (Davis et al., 1993), the Test of Functional Health Literacy in Adults (Parker et al., 1995), and the European Health Literacy Survey (Sørensen et al., 2013), most focus on assessing respondent comprehension of medical terminology and health-related scenarios, with limited emphasis given to HPV-prevention information. Research on HPV infection has focused predominantly on topics related to cervical cancer and the willingness of individuals to receive the HPV vaccination (Johnson et al., 2022; K.-M. Zhang et al., 2024), with little attention given to HPV prevention-related health literacy. Therefore, this study was designed to establish and test the validity and reliability of the HPV Prevention Health Literacy Scale (HPV-HLS). Using comprehensive psychometric testing, this measurement tool was designed to serve as a valuable resource for evaluating the effectiveness of HPV prevention and intervention measures.

## Methods

### Design

A cross-sectional design was used to assess the validity and reliability of the HPV-HLS between November 2021 and December 2022.

### Item Construction

The initial definitions and item-generation procedure for the HPV-HLS were informed by the authors’ previous qualitative research (Lai et al., 2024) and by the health literacy framework proposed by Nutbeam (1999). Using a qualitative descriptive approach, in-depth interviews were conducted with 26 college students to explore their perspectives on health literacy and HPV prevention. This resulted in an initial pool of 49 scale items. Five experts, including a professor specializing in HPV-related cancer nursing, a professor experienced in scale construction, and three senior attending physicians in obstetrics, gynecology, and pediatrics, evaluated the relevance, accuracy, and appropriateness of each item’s wording. Using the item content validity index (I-CVI) and scale content validity index/average (S-CVI/Ave) to assess content validity (Cho & Kim, 2024; Liao et al., 2025). Following previous studies, an I-CVI score of ≥ .78 and an S-CVI/Ave score of ≥ .90 were used as the criteria (Polit et al., 2007). Using a four-point Likert scale, the experts rated each item, and the ratings were used to calculate item and scale content validity indices. Based on the comments and suggestions of the experts, the authors, through mutual discussion, deleted the five items with scores ≤ .78 (i.e., 1, 15, 29, 47, 48) due to overlapping meanings, irrelevance, or inappropriateness. Some of the remaining items were then reworded for clarity. The remaining 44 items had I-CVI scores ranging from .80 to 1.00 and an S-CVI/Ave score of .90.

### Item Analysis

Scale development followed the procedures suggested by Tu (2023). In the pilot study, 30 college students who met the inclusion criteria completed the 44-item HPV-HLS. Each scale item is rated on a 4-point Likert scale from 1 (*very difficult*) to 4 (*very easy*), with higher scores indicating higher levels of HPV prevention health literacy.

The item analysis, based on independent-samples *t* tests comparing high-scoring and low-scoring groups for each item, showed *p* values > .05, resulting in the removal of 7 items (i.e., 3, 4, 7, 8, 9, 19, 31). The corrected item-total correlation coefficients ranged from .54 to .83, and the factor loadings ranged from .22 to 1.08 (Table 1). The remaining 37 items were subsequently tested for reliability and validity in the formal study.

**Table 1:** Scale Item Analysis

Item	*t*	Factor Loading	*r*	Retained Item
2	3.12*	.45	.58	√
3	2.00	.88	.53	×
4	2.08	.59	.52	×
5	2.76*	.89	.57	√
6	3.61**	1.08	.59	√
7	2.14	.63	.51	×
8	1.80	.62	.35	×
9	1.85	.42	.44	×
10	3.86**	.41	.64	√
11	2.69*	.51	.58	√
12	3.86**	.32	.63	√
13	2.39*	.34	.55	√
14	6.18***	.60	.77	√
16	8.28***	.65	.83	√
17	4.58**	.32	.60	√
18	3.86**	.55	.57	√
19	0.78	.42	.33	×
20	2.97*	.41	.59	√
21	5.60***	.51	.81	√
22	7.90***	.67	.78	√
23	4.29**	.51	.68	√
24	3.79**	.69	.72	√
25	3.99**	.59	.63	√
26	7.94***	.53	.72	√
27	3.90**	.51	.61	√
28	5.15***	.56	.72	√
30	3.81**	.44	.64	√
31	1.82	.44	.44	×
32	4.66**	.55	.61	√
33	3.60**	.49	.67	√
34	5.23**	.44	.75	√
35	7.00***	.22	.75	√
36	5.46***	.49	.70	√
37	2.18*	.71	.54	√
38	4.29**	.49	.69	√
39	4.66**	.46	.72	√
40	4.66**	.62	.69	√
41	3.60**	.59	.63	√
42	2.58*	.28	.64	√
43	2.58*	.33	.60	√
44	4.25**	.37	.69	√
45	7.00***	.65	.82	√
46	3.00*	.25	.67	√
49	3.99**	.44	.71	√

*Note. t*-values represent the results of independent *t* tests between high-scoring and low-scoring groups for each item; *r* = corrected item-total correlation coefficients; ‘√’ indicates the item was selected.

*p < .05. **p < .01. ***p < .001.

### Participants

This study was conducted at two universities in northern Taiwan and one in central Taiwan, encompassing both public and private schools in urban areas. Convenience sampling was supplemented by snowball sampling to enhance participant recruitment. The inclusion criteria comprised college students aged 20–25 years old. Individuals with no exposure to information regarding HPV were excluded. According to Beavers et al. (2013), a minimum sample size of 150 is necessary for exploratory factor analyses (EFAs) to ensure stable statistical parameter estimates. Confirmatory factor analysis (CFA) was performed using the 37-item scale following expert content validation and item analysis. The sample size was determined using the general rule of factor analysis (Hoyle, 2000), which recommends at least 5 respondents per item and a minimum sample size of 200. Three hundred seventy-two valid responses were collected in this study, with computer-assisted random sampling used to select 150 for EFA and 222 for CFA.

### Ethics and Data Collection

This study was approved by the Research Ethics Committee (202108ES008). With support from the faculty members at the study sites, the investigator presented the study details to the college students in classroom settings. Their right to withdraw from the study without impact on their academic standing or learning privileges was emphasized. Those students who expressed interest in participating were provided a link to an online electronic questionnaire. The initial questionnaire screen included consent-to-participate information, and participants were asked to click “I agree” before proceeding. Completed questionnaires were submitted directly to the researchers via the online platform. On average, the participants spent approximately 10 to 15 minutes completing the questionnaire. Current participants were also invited to share the online questionnaire link with eligible college peers who are interested in participating. To minimize duplicate responses and ensure data integrity, IP tracking was implemented, and participants were directed to complete the survey only once. All research data were anonymized, coded, stored in password-protected computer files, and kept confidential to safeguard participant privacy.

### Data Analysis

SPSS 26.0 (IBM Corp., Armonk, NY, USA) was used in this study to conduct demographic analyses, EFA, and reliability analyses. A total of 150 valid responses were selected for EFA. Bartlett’s test of sphericity and the Kaiser-Meyer-Olkin (KMO) measure were employed to assess sampling adequacy (Cho & Kim, 2024), with a KMO index > .70 and a significant result on Bartlett’s test of sphericity (*p* < .001) indicating the study data as suitable for EFA (Pett et al., 2003). Principal component analysis was used for factor extraction, with the number of factors determined by eigenvalues >1. The varimax method was applied for orthogonal rotations, with factor loadings > .40 used to determine item contributions to the factors (Costello & Osborne, 2005). Subsequently, oblique rotations of the varimax method were implemented to achieve a more appropriate level of convergence. After the items were removed, another round of EFA was conducted to determine factor and item contributions.

AMOS 26.0 software (IBM Corp, Armonk, NY, USA) was used in this study to conduct the CFAs. Model goodness-of-fit was assessed based on the following criteria: χ^2^ (*p* > .05); goodness-of-fit index (GFI) > .80; comparative fit index (CFI) and Tucker-Lewis index (TLI) > .90; root mean square error of approximation (RMSEA) < .08; standardized root mean square residual (SRMR) < .05; and χ^2^ degree of freedom (χ^2^ */df*) < 3 (Doll et al., 1994; Hair et al., 2010; Tabachnick & Fidell, 2013). In addition, indicators of convergent validity included factor loadings > .50, composite reliability (CR) > .70, and average variance extracted (AVE) > .50 (Hair et al., 2010). The AVE for each factor exceeded the square of its correlation coefficient (Cheung et al., 2024), supporting good convergent and discriminant validity for the proposed scale.

**Figure 1:**
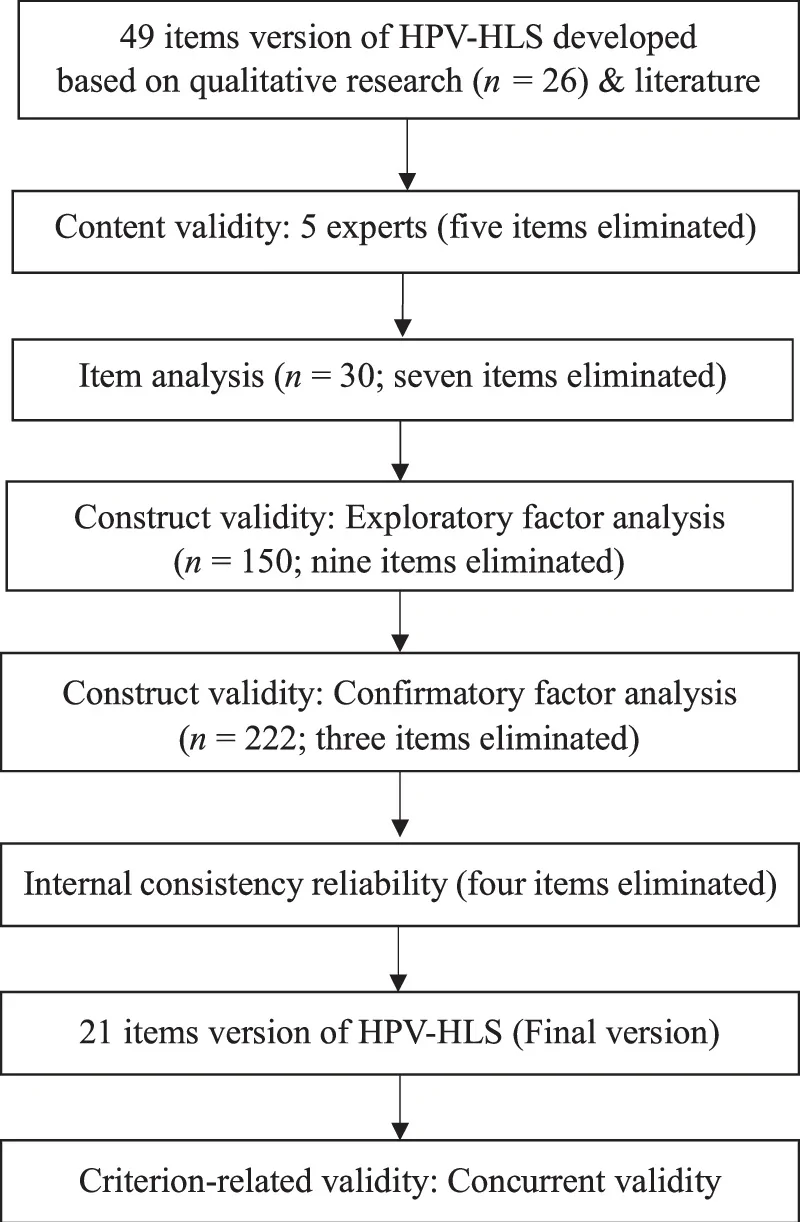
Psychometric Testing Process Used for the Human Papillomavirus Prevention Health Literacy Scale for College Students

Finally, internal consistency reliability was assessed. Concurrent validity was examined by analyzing the relationship between the Mandarin Multidimensional Health Literacy Questionnaire (MMHLQ; Wei et al., 2017) and HPV-HLS. The 20-item MMHLQ was designed to evaluate health literacy across the five domains of accessing, understanding, appraising, applying, and communicating health information. Scoring is based on a four-point Likert scale, ranging from 1 (*very difficult*) to 4 (*very easy*), with higher scores indicating higher levels of health literacy. The Cronbach’s alpha coefficient for the MMHLQ is .94, with a test–retest reliability of .85. MMHLQ is frequently employed as a health literacy assessment tool for older adults and patients with chronic diseases (Chiou et al., 2022; Shih et al., 2021). Its usage in this study was authorized by the original author. The process used to psychometrically test the HPV-HLS is illustrated in Figure 1.

## Results

### Participant Characteristics

Three hundred seventy-two college students participated in this study (166 male and 206 female). The mean participant age was 20.38 (*SD* = 3.54, range 18–25) years. Among these, 113 (31.8%) reported being sexually experienced, with the number of sexual partners ranging from 1 to 10 (*M* = 3.01, *SD* = 2.69). Age at first intercourse ranged from 13 to 22 (*M* = 17.85, *SD* = 1.69) years. An overview of the sample’s demographic characteristics is given in Table 2.

**Table 2:** Descriptive Statistics for Demographic Characteristics (*N*=372)

Characteristic	Total	EFA Sample	CFA Sample
	(*n*=372)	(*n*=150)	(*n*=222)
	*M*±*SD/ n* (%)	*M*±*SD/ n* (%)	*M*±*SD/ n* (%)
Age (years)	20.38±3.54	20.79±4.49	19.97±2.58
Gender			
Male	166 (44.5)	66 (44.0)	100 (45.0)
Female	206 (55.5)	84 (56.0)	122 (55.0)
Sexual orientation			
Heterosexual	327 (87.5)	128 (85.3)	199 (89.6)
Homosexual	14 (3.8)	6 (4.0)	8 (3.6)
Bisexual	31 (8.7)	16 (10.7)	15 (6.8)
Degree course			
Medicine	104 (28.8)	50 (33.3)	54 (24.3)
Nursing	140 (36.4)	45 (30.0)	95 (42.8)
Health Sciences	86 (23.9)	42 (28.0)	44 (19.8)
Humanities and Management	42 (10.9)	13 (8.7)	29 (13.1)
Sexual experience			
Yes	113 (31.8)	59 (39.3)	54 (24.3)
No	259 (68.2)	91 (60.7)	168 (75.7)
Age at first intercourse	17.85±1.69	17.78±1.73	17.91±1.65
No. sexual partners	3.01±2.69	3.17±2.99	2.85±2.39
HPV vaccination status			
Vaccinated	123 (32.2)	42 (28.0)	81 (36.5)
Nonvaccinated	249 (67.8)	108 (72.0)	141 (63.5)
Prior experience being consulted about HPV infection			
Yes	107 (28.2)	38 (25.3)	69 (31.1)
No	265 (71.8)	112 (74.7)	153 (68.9)

*Note.* CFA = confirmatory factor analysis; EFA = exploratory factor analysis; HPV = human papillomavirus.

### Construct Validity

#### Exploratory Factor Analysis

The results of Bartlett’s test of sphericity for the 37-item HPV-HLS yielded statistically significant findings (χ^2^ = 5608.231, *p* < .001), indicating a significant correlation among the extracted factors. In addition, two factors with KMO values of .93 were identified based on their eigenvalues exceeding 1 and the scree plot. Principal component analysis with orthogonal rotations was then employed, leading to the deletion of eight items (i.e., 5, 30, 33, 34, 35, 36, 37, 38) due to factor loadings below .40. A second EFA was then conducted, resulting in the deletion of item 14 due to a factor loading below .40, which led to a revised total of 28 items for the scale.

After conducting EFA with oblique rotation, the factor loadings ranged between .459 and .996. Factor 1, comprehension and evaluation of general information, comprised 19 items (2, 6, 10, 11, 12, 13, 16, 17, 18, 20, 21, 22, 23, 24, 25, 26, 27, 28, 32) and accounted for 51.58% of the variance. Factor 2, seeking information from others, encompassed 9 items (39, 40, 41, 42, 43, 44, 45, 46, 49) and explained 8.72% of the variance. Together, these two factors explained 60.30% of the total variance. Factor 1 addresses the ability of college students to comprehend fundamental information about HPV prevention, including medical terminology, transmission routes, preventive measures, and treatment, and their ability to evaluate the authenticity and quality of this information based on personal experiences and values, which facilitates the adoption of proactive measures such as practicing safe sex and getting vaccinated. Factor 2 addresses the level of college student engagement in communication and discussion with various individuals, including relatives, friends, and health care professionals, which is associated with the ability to acquire new knowledge and rectify misconceptions. These two factors were named based on the theoretical framework of health literacy and the thematic content of the retained items. Specifically, Factor 1 reflects functional and critical health literacy competencies, while Factor 2 corresponds to interactive health literacy. The factor names were selected to represent the conceptual meaning underlying these domains.

#### Confirmatory Factor Analysis

**Figure 2:**
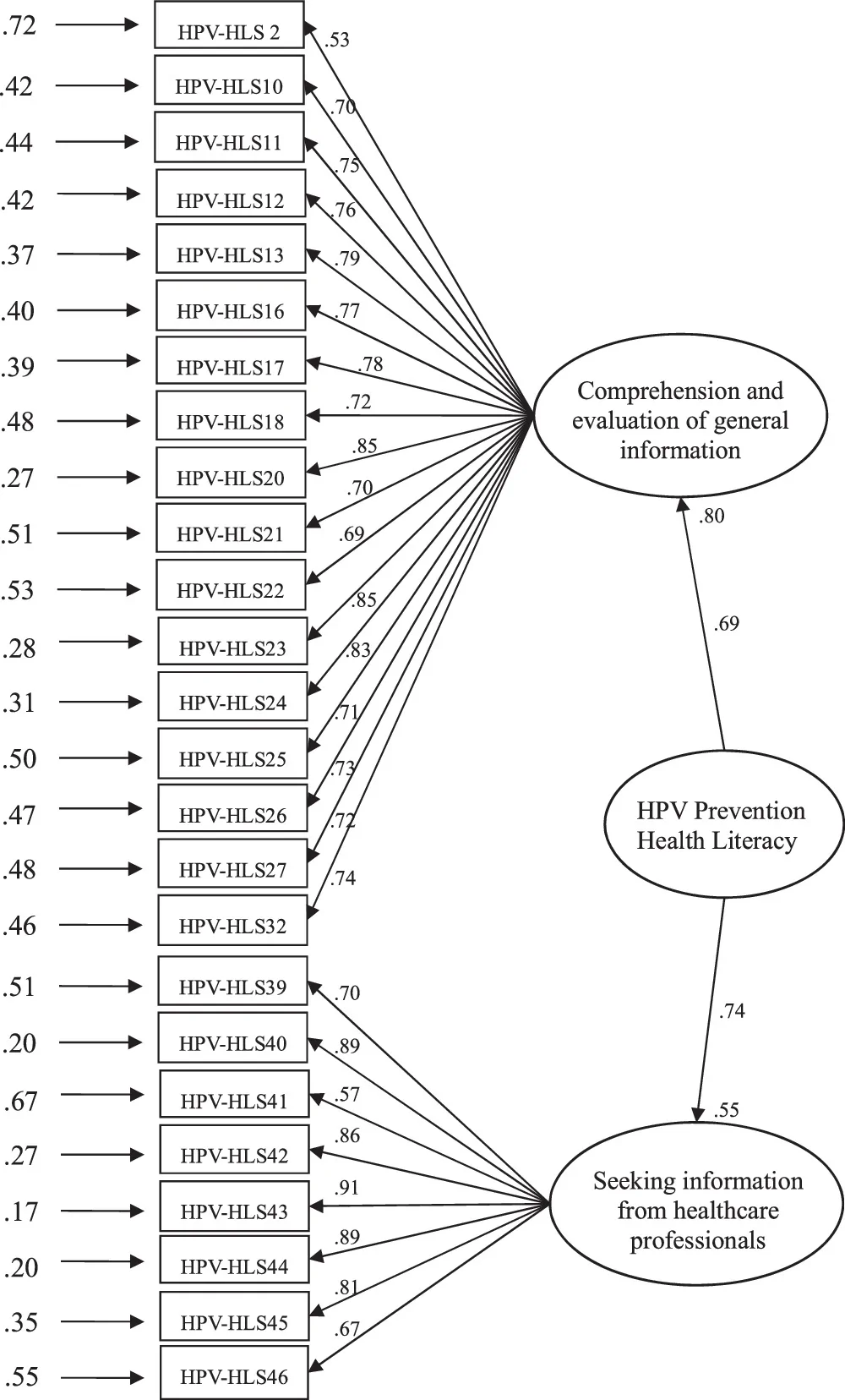
Factor Loadings for the Confirmatory Factor Analysis of the Human Papillomavirus Prevention Health Literacy Scale *Note.* HPV-HLS = Human Papillomavirus Prevention Health Literacy Scale.

CFA was conducted using the 28 items derived from EFA. A second-order two-factor structure model was specified. Two factors were employed in the first-order factor structure as latent variables, with their respective question items serving as measurement variables. The second-order factor structure integrated HPV prevention health literacy as a higher-order latent variable, with the two first-order factors representing the lower-order latent variables. Items 6 and 28, which exhibited standardized factor loadings below .50, were removed, leaving 26 remaining scale items. To improve goodness of fit, adjustments were made to modify the standardized residuals. Following a recommendation by Hair et al. (2010), standardized residual values exceeding 2.58 prompted the consideration to modify the path relationships between variables. In the analysis results, standardized residual values exceeding 2.58 were observed between item 49 and items 10 (3.559), 11 (3.150), 12 (2.952), 13 (2.696), 16 (2.759), 18 (2.653), 20 (3.410), 21 (2.758), 22 (2.886), 23 (3.089), 25 (2.837), 26 (3.068), and 32 (2.825). Therefore, item 49 was removed, leaving 25 scale items (Figure 2). In terms of overall model fit, although the χ^2^ analysis yielded a significant result (χ^2^ = 606.190, *p* < .001), the favorable χ^2^ */df* ratio of 2.21 (<3) indicated a robust fit. Moreover, all model fit indicators demonstrated good performance (GFI = .81, CFI = .92, TLI = .91, RMSEA = .07, SRMR = .02). Notably, all factor loadings in the model exceeded .50 and the CR for each factor exceeded .70 (comprehension and evaluation of general information: .96; seeking information from others: .93). Moreover, the AVE exceeded .50 for both factors (comprehension and evaluation of general information: .56; seeking information from others: .64), with the AVE of the two factors exceeding the square of their correlation coefficient. These findings indicate the scale reflects excellent convergent and discriminant validity. The Pearson correlation coefficient between the two factors was statistically significant (*r* = .66, *p* < .001).

### Internal Consistency Reliability

Using the responses to the 25 items provided by the 222 participants used in the CFA, the Cronbach’s alpha coefficients of the HPV-HLS and its subscales were examined to assess internal consistency. The Cronbach’s alpha value for the 17-item comprehension and evaluation of general information subscale was .954 and, after removing item 2, rose to .955. Similarly, the Cronbach’s alpha value for the 9-item seeking information from others subscale was .928 and, after removing three items (39, 41, 45) and renaming the subscale to “seeking information from health care professionals,” rose to .939. Thus, the final version of the scale incorporates a total of 21 items (Appendix, Supplemental Digital Content 1, https://links.lww.com/JNR/A15). The Cronbach’s alpha value for the overall scale of .959 indicates excellent reliability (Table 3).

**Table 3:** Reliability Testing

Item	*M*±*SD*	Cronbach’s α Before Item Deleted	Cronbach’s α After Item Deleted	Retained Item
Factor 1: Comprehension and evaluation of general information		.954	.955	
2. Searching for information on HPV prevention from the internet is…	3.33±0.65	.955		×
10. Understanding the content of HPV prevention information from different sources is…	2.97±0.63	.951	.952	√
11. Understanding the routes of transmission for HPV infection is…	3.15±0.64	.951	.952	√
12. Understanding the preventive measures for HPV is…	3.09±0.65	.951	.952	√
13. Understanding HPV screening methods is…	2.91±0.66	.951	.951	√
16. Understanding the impacts of various HPV vaccine types (valence) on preventing disease in different sexes is…	2.90±0.72	.951	.952	√
17. Understanding the health benefits of HPV vaccination is…	3.17±0.68	.951	.952	√
18. Understanding the health benefits of HPV vaccination in different sexes is…	2.71±0.74	.952	.953	√
20. Understanding HPV prevention information provided by health care professionals is…	2.95±0.66	.950	.951	√
21. Identifying the accuracy of information on HPV is…	2.80±0.77	.952	.953	√
22. Determining the completeness of HPV prevention information is…	2.80±0.77	.953	.954	√
23. Evaluating whether HPV infection prevention information applies to me is…	2.96±0.64	.950	.951	√
24. Determining the consistency of the HPV information obtained is…	2.82±0.68	.950	.951	√
25. Determining the credibility of the HPV information obtained is…	3.00±0.78	.952	.953	√
26. Determining if I need the HPV vaccine is…	2.88±0.75	.952	.953	√
27. Discerning which type (valence) of HPV vaccine is suitable for me based on the obtained information on HPV prevention is…	2.88±0.72	.952	.953	√
32. Using HPV-related information to help me choose the appropriate HPV type (valence) vaccine is…	2.80±0.71	.952	.952	√
Factor 2: Seeking information from health care professionals		.928	.939	
39. Expressing my doubts about HPV information to health care professionals is…	2.85±0.59	.925		×
40. Asking health care professionals about prevention measures for HPV infection is…	3.00±0.63	.910	.923	√
41. Asking health care professionals about information on the types (valence) of HPV vaccines is…	3.29±0.63	.934		×
42. Expressing a request for HPV screening with health care professionals is…	2.98±0.64	.915	.927	√
43. Asking health care professionals about treatments for HPV-related diseases is…	2.99±0.65	.910	.918	√
44. Confirming the accuracy of my understanding of HPV infection prevention information with health care professionals is…	3.00±0.64	.911	.921	√
45. Discussing HPV issues with health care professionals is…	2.88±0.68	.917	.937	√
46. Discussing treatment plans with health care professionals after an HPV infection is diagnosed is…	3.09±0.63	.927		×
Overall scale		.959	.959	

*Note.* HPV = human papillomavirus. ‘√’ indicates that the item was selected.

### Concurrent Validity

The results of the CFA demonstrated a significant association between the MMHLQ and HPV-HLS (*r* = .68, *p* < .001), indicating the robust concurrent validity of the HPV-HLS.

## Discussion

In this study, a scale was developed using qualitative interviews and a thorough review of relevant literature. Psychometric testing included expert content validation, item analysis, construct validation, assessment of convergent and discriminant validity, internal consistency reliability evaluation, and concurrent validity assessment. This effort resulted in the 21-item HPV-HLS, which incorporates the two distinct subscales of comprehension and evaluation of general information (16 items) and seeking information from health care professionals (5 items).

The first subscale addresses the ability of college students to understand HPV prevention information and to evaluate the authenticity and quality of this information based on their personal experiences and values, which relate positively to adopting protective and preventive measures. This concept aligns with the established definitions of functional literacy and critical health literacy (Nutbeam, 1999, 2000). The second subscale (seeking information from health care professionals) deviates slightly from Nutbeam’s definition of interactive health literacy, which encompasses interactions with relatives, friends, and health care professionals (Nutbeam, 1999). The primary focus of the subscale in the proposed scale is on communication and discussion with health care professionals. This aspect of health literacy aligns with the authors’ previous qualitative interviews (Lai et al., 2024), in which many college students expressed a preference for information from health care professionals rather than relatives or friends. They perceived information provided by professionals to be more reliable and credible, and to facilitate the acquisition of new knowledge, the receipt of valuable feedback, and the clarification of misconceptions through communication and interaction with health care professionals. Benavidez et al. (2020) noted that seeking information from relatives and friends regarding HPV infection may result in misinformation due to their lack of relevant domain expertise, which can further deepen misunderstandings among young adults.

Research has supported the critical role of understanding preventive health information in enabling college students to make informed decisions about their health, thereby facilitating effective health promotion and maintenance (Albright & Allen, 2018). Insufficient health literacy, characterized by poor understanding of health information and ineffective communication with health care professionals, may adversely impact health outcomes, leading to an increased incidence of infectious diseases and reduced use of preventive care services, which can consequently drive the overuse of medical resources (Deek et al., 2025; Jang et al., 2024). The scale developed in this study offers an effective tool for assessing HPV infection prevention-related health literacy, particularly among college students seeking to acquire knowledge from professionals on this topic.

The results of the extreme value test of the HPV-HLS items in the item analysis indicate that the scale provides a high degree of discrimination. Furthermore, the single-item total scale correlation test showed high homogeneity within each subscale. Moreover, the results of both the EFA and CFA further substantiated the construct validity of the HPV-HLS. The EFA findings indicate that all items met the criterion of a factor loading of at least .50 (Hair et al., 2010). Similarly, the CFA outcomes met the recommended standards for assessing goodness-of-fit (Doll et al., 1994; Hair et al., 2010; Tabachnick & Fidell, 2013) and exhibited excellent convergent and discriminant validity, with factor loadings exceeding .50, CR exceeding .70, and the AVE greater than .50 (Hair et al., 2010). The second-order factor model supported the presence of a higher-order health literacy construct specific to HPV infection prevention, extending beyond the two individual factors. The Cronbach’s alpha coefficient for the entire scale of .96 indicates outstanding internal consistency (Nunnally & Bernstein, 1994). Concurrent validity testing, using Pearson’s correlation, between the proposed HPV-HLS and the MMHLQ revealed a significant correlation (*r* = .68, *p* < .001). Based on the above, the HPV-HLS demonstrated robust reliability and validity across multiple analyses, affirming its suitability as a comprehensive tool for assessing health literacy related to HPV infection prevention.

### Implications for Nursing Practice

Understanding and addressing college students’ health literacy regarding HPV infection prevention is crucial for health care providers and educators. The HPV-HLS developed in this study provides an effective tool for assessing college students’ HPV prevention health literacy, with significant implications for health education and school nursing practice. The proposed scale demonstrated good validity and reliability and was user-friendly, requiring only a few minutes to administer and score. The four main implications of the proposed scale for health education and school nursing practice include (a) the HPV-HLS enables objective and rapid measurement of college students’ HPV prevention health literacy levels, allowing health care providers and educators to accurately understand students’ literacy in this area, with the results available to inform effective health education planning initiatives; (b) the HPV-HLS assists health care providers and educators in identifying student subgroups with lower health literacy, enabling targeted interventions and efficient resource allocation to enhance the effectiveness of health education efforts; (c) the HPV-HLS provides a tool for health care providers and educators to identify at-risk students early on. By pinpointing groups with lower literacy levels, health care providers and educators can provide tailored support and resources to help students improve their understanding of HPV-related health issues; and (d) the HPV-HLS may be used further to evaluate the impact of HPV-related curricula or educational activities, enabling health care providers and educators to continually adjust educational strategies to improve the overall effectiveness of HPV prevention education. Data generated by the HPV-HLS can provide health care providers and educators with a reliable basis for promoting health literacy.

### Strengths and Limitations

The strengths of this study lie in the use of both qualitative and quantitative methodologies to develop the scale items and in the comprehensive psychometric evaluation. The EFA facilitated item reduction and factor identification, while the CFA validated the factors. Furthermore, their application in a two-step analysis approach on two different samples helped mitigate the risk of Type I error (Kyriazos, 2018). In addition, participant recruitment encompassed students from both public and private institutions and spanned different genders, departments, and sexual experience statuses. Data diversity enhances the generalizability of findings. Regarding limitations, the test–retest reliability of the HPV-HLS could not be assessed due to participants’ anonymity. Consistent with Lin et al. (2024), this study used a cross-sectional design with anonymous online questionnaires, limiting the ability to assess temporal stability within the same participant group. The authors intend to further validate the test–retest reliability of the HPV-HLS, including college students with no prior exposure to HPV-related information, in future research. Random sampling across various geographic regions will also be conducted to enhance the applicability and generalizability of HPV-HLS.

### Conclusions

In this study, the HPV-HLS, developed by the authors through qualitative interviews and a literature review, demonstrated strong reliability and validity. This scale holds strong potential as a practical self-report measurement tool for evaluating health literacy related to HPV infection prevention and may be applied as both a clinical assessment tool and a measure of intervention effectiveness.

## Supplementary Material
